# Fasudil improves endothelial dysfunction in rats exposed to chronic intermittent hypoxia through RhoA/ROCK/NFATc3 pathway

**DOI:** 10.1371/journal.pone.0195604

**Published:** 2018-04-11

**Authors:** Jie-Ru Li, Ya-Shuo Zhao, Yue Chang, Sheng-Chang Yang, Ya-Jing Guo, En-Sheng Ji

**Affiliations:** 1 Department of Physiology, Hebei University of Chinese Medicine, Shijiazhuang, Hebei, P.R. China; 2 Scientific Research Center, Hebei University of Chinese Medicine, Shijiazhuang, Hebei, P.R. China; Center for Cancer Research, UNITED STATES

## Abstract

Endothelial dysfunction is one of the main pathological changes in Obstructive sleep apnoea (OSA). The Rho kinase (ROCK) pathway is associated with endothelial dysfunction. However, the interaction between ROCK and nuclear factor of activated T cells isoform c3 (NFATc3) in the development of this pathological response under chronic intermittent hypoxia (CIH) is unclear. To simulate the OSA model, we established a moderate CIH rat model by administering the fraction of inspired O_2_ (FiO_2_) from 21% to 9%, 20 times/h, 8 h/day for 3 weeks. Fasudil (ROCK inhibitor, 8 mg/kg/d, i.p.) was administrated in the rats exposed to CIH for 3 weeks. Our results demonstrated that CIH caused significantly endothelial dysfunction, accompanying with increased ET-1 level, decreased eNOS expression and NO production, which reduced ACh-induced vascular relaxation responses. Moreover, RhoA/ROCK-2/NFATc3 expressions were up-regulated. Fasudil significantly improved CIH induced endothelial dysfunction. Data suggested that the ROCK activation is necessary for endothelial dysfunction during CIH.

## Introduction

Obstructive sleep apnoea (OSA) is a complete or partial airway obstruction, resulting in significant physiological disturbance with multiple clinical influences [1]. The aetiology of OSA is multifactorial, and it’s reported the patients exhibited snoring at night, headache while waking up, sleepiness in the daytime and decreasing cognitive performance in clinically [2]. Recent epidemiological studies have revealed that the OSA prevalence was approximately 3–7% in men and 2–5% in women [3, 4]. Studies have shown that OSA could increase the prevalence and incidence of cardiovascular diseases [5, 6], such as atherosclerosis, coronary heart disease, heart failure, arrhythmia and hypertension.

There may be many possible influencing factors linking OSA with cardiovascular diseases; however, the specific mechanism has not been fully elucidated. Some studies have shown that endothelial dysfunction, as part of the pathogenesis of cardiovascular diseases, was significantly correlated with OSA [7]. The vascular endothelium participates in the release of multiple vasoactive factors, including the vasodilator nitric oxide (NO) and the vasoconstrictor endothelin-1 [8], which played a major role in the pathogenesis of cardiovascular problems such as atherosclerosis, systemic and pulmonary hypertension, and cardiomyopathies [9]. OSA is characterized by chronic intermittent hypoxia (CIH) and CIH could trigger systemic endothelial dysfunction, which suggested that regulating the ability of vascular tone and repair capacity in the endothelium were weakened [10]. In rats exposed to CIH, the circulating endothelin-1 (ET-1) level and the susceptibility of vasoconstriction to ET-1 were enhanced [11, 12], and vascular NO bioavailability was decreased [10].

The small GTP-binding protein RhoA and its downstream target, Rho kinase (ROCK), have recently been studied in the cardiovascular field. Activated ROCK was associated with atherosclerosis and arterial hypertension in experimental rat models [13, 14] and clinical patients [15, 16]. Studies have shown that the ROCK inhibitor (fasudil) treatment could decrease the atherosclerosis lesions through decreasing the thickness of arterial intima medial and macrophage accumulation [17]. On the other hand, nuclear factor of activated T cells isoform c3 (NFATc3) belongs to the NFAT transcription factors family that have the nature of calcineurin-dependent nuclear translocation. It is important to note that the activation of Rho/ROCK is involved with pathways that regulate NFAT activity [18]. Some studies have demonstrated that NFATc3 was related to pulmonary hypertension induced by CIH in mice [19, 20], however, the mechanism by which RhoA/ROCK/NFATc3 mediates CIH-induced endothelial dysfunction has not been fully clarified.

In the study, we imitated OSA using a rat model of CIH to investigate the role of ROCK, and detected whether CIH might affect RhoA/ROCK/NFATc3 mediated endothelial dysfunction in aortas. Therefore, in this study we hypothesized that the fasudil treatment could inhibit the CIH-induced endothelial dysfunction in rats. Further, we investigated if fasudil would restore endothelial dysfunction induced by CIH and its mechanisms.

## Materials and methods

### Experimental animals

#### Ethical approval

All procedures were performed based on the National Institutes of Health Guide for the Care and Use of Laboratory Animals and were authorized by the Animal Care and Use Committee of Medical Ethics of Hebei University of Chinese Medicine (approval number: HEBUCM-2014-07; approval date: July 01, 2014). Adult male Sprague-Dawley rats (190–220g) were purchased from the Hebei Experimental Animal Center (Shijiazhuang, China). All rats were given free access to food and water, housed under constant temperature and controlled illumination. All rats were allowed to adapt to their living conditions for at least 7 days before experiment.

#### The test of fasudil

To assess the effect of fasudil on the endothelial function, an experiment was firstly performed. Fasudil was purchased from Cheng Tian Heng Chuang Biological Technology Company. Twelve rats were randomly divided into control group and fasudil group. Rats in fasudil group were administered with fasudil (8 mg/kg/day, i.p., once a day, at 9 a.m.) for 4 weeks. While, the rats in control group were received an equal volume of normal saline. All rats were observed daily for general status, behaviour, morbidity and mortality. Body weight (BW), tail-cuff systolic blood pressure (SBP) and heart rate (HR) were measured at the beginning of the study and once a week thereafter. Food consumption was recorded weekly. 24 h after the last administration of the drug, two groups of rats were sacrificed. Their blood samples were used for blood biochemistry, and their aortas were dissected for histopathological analysis.

#### Experimental grouping and CIH model

SD rats (n = 36) were randomly divided into three groups (n = 12, for each group): normoxia control group (Normoxia), CIH model group (CIH) and fasudil-treated CIH model group (CIH + Fa). These rats were housed in special hypoxic chambers with a controlled gas delivery system that monitored the flow of air, nitrogen and oxygen into the chambers. The fraction of inspired oxygen (FiO_2_) provided to the chambers for the CIH and the CIH + Fa groups declined from 21% to 9% for 90 s, and then gradually increased to 21% with re-oxygenation in the subsequent 90 s period. The exposure cycle was repeated every 3 min for 8 h/day, for 3 weeks. In addition, the rats in the CIH + Fa group were also successively given fasudil (8 mg/kg/day, i.p., once a day) for 3 weeks. Rats in the Normoxia and CIH groups were injected with an equal volume of normal saline at the same time points.

#### Tissue and blood sample processing

After 21 days, the rats were fasted for one night. They were weighed and anaesthetized with pentobarbital (100 mg/kg, i.p.). Half of the rats in each group (n = 6, for each group) were used for the ACh-induced vascular relaxation responses study, and the other half of rats in each group (n = 6, for each group) were used for blood and tissue determination. Blood samples were obtained from the femoral aorta, and the serum was separated and collected for biochemical analysis. At the same time, thoracotomy and thoracic aorta tissues were collected. Collected thoracic aorta tissues were used for western blot analysis, nitrate reductase method detection and histological analysis.

### Evaluation of vasodilator responses

#### The isolation of aortic vessels

The thoracic aortas were excised and immediately placed in 4°C physiological saline solution (PSS, pH 7.4, 133.1 mM NaCl, 4.7 mM KCl, 0.61 mM MgSO_4_, 1.3 mM NaH_2_PO_4_, 16.7 mM NaHCO_3_, 2.5 mM CaCl_2_, 7.6 mM Glucose). The thoracic aortas were carefully isolated and cut into 3 mm rings. Then, vessel endothelia were stripped mechanically by inserting watchmaker’s forcep tips into the vascular lumen and the vessel was repeatedly rotated on saline-saturated filter paper [21].

#### Detection of vasodilator responses

Rings of arteries were suspended horizontally in organ chambers filled with 6 ml PSS sustained at 37°C and inflated with 95% O_2_ and 5% CO_2_. Two stainless steel wires passed through the vessel ring lumen; one was fixed to the bottom of the organ chamber, and the other was attached to a strain gauge. Isometric tension was measured with a Power-Lab/8sp recording and analysis system (Model ML785; AD Instruments, Castle Hill, NSW, Australia).

Each vascular ring was progressively extended to its optimal resting tension until the contraction force of the vascular ring in 70 mM KCl reached a plateau; the optimal resting tension of rat thoracic aortas was 1.5 g. Each ring was equilibrated for 1 hour. After equilibration, viability was verified by contraction with 10^−6^ M phenylephrine (PE, Sigma Chemical, St. Louis, MO), and vasodilator responses to vasodilator acetylcholine (ACh, Sigma, 10^−6^ M) and sodium nitroprusside (SNP, Sigma, 10 ^−6^ M) were tested.

Endothelium denudation was confirmed as a < 5% relaxation response to 10^−6^ M ACh in rings preconstricted with 10^−6^ M PE. To determine whether CIH affected vasodilator responses, basal aortic tone was tested by pre-incubating the rings with 10^−6^ M PE and examed relaxation responses to 10^−6^ M ACh and 10^−6^ mol/L SNP. Relaxation responses to ACh and SNP are expressed as a percentage of the PE-induced tone.

### Measurement of ET-1 levels in serum

The serum ET-1 concentration was measured by radioimmunoassay (RIA) as per the instructions of the kit (SenBeiJia Biological Technology, Nanjing, China). By using the non-equilibrium method, we made a standard curve and the content of samples was calculated.

### Measurement of NO content in aortic tissue and serum

The NO content was defined by measuring total nitrate and nitrite concentrations (Nitric Oxide Assay Kit: Nanjingjiancheng Biological Engineering Institute, Nanjing, China). This assay determined total content of NO based on the enzymatic conversion of nitrate to nitrite by nitrate reductase. The reaction was followed by the colourimetric detection of nitrite as an azo dye product of the Griess reaction. The absorbance of the compound at 550 nm was detected with a microplate reader.

### Histological analysis

The aortic tissues removed from all rats were fixed in 4% paraformaldehyde for 48 h. After fixation, the tissues were dehydrated in alcohol gradient and embedded in paraffin. Tissue slices were cut at 5 μm thickness and stained with haematoxylin and eosin (H&E) for histological analysis. Each section was observed under 10 × 40 light microscopic fields with an optical microscope (Olympus Japan Co., Tokyo, Japan).

### Western blot

Frozen thoracic aortic samples were homogenized and lysed using RIPA lysis buffer with a mixture of protease inhibitors. The homogenates were centrifuged at 12,000 g at 4°C for 20 min, and the supernatant was collected. The total protein concentration was measured with a BCA Protein Assay Kit (Aidlab Biotechnologies Company, China). Then, 40 μg of protein was added into sodium dodecyl sulfonate (SDS)-polyacrylamide gels and transferred to polyvinylidene difluoride membranes (Millipore, Billerica, MA, USA). After blocking with non-fat milk, the blots were incubated overnight at 4°C with primary antibodies for ET-1 (Abcam, ab2786, 1:600), ROCK-2 (Abcam, ab71598, 1:600), anti-eNOS (Abcam, ab50010, 1:600), anti-p-eNOS Ser_1177_ (Abcam, ab195944, 1:600), anti-RhoA (Abcam, ab187027, 1:3000), anti-MYPT (Cell Signaling, 2634p, 1:1000), anti-p-MYPT (Cell Signaling, 4563p, 1:1000), anti-NFATc3 (Novus, NB100-92190, 1:500) and anti-β-actin (Sigma, A5316, 1:1000). MYPT and p-MYPT protein were measured to indirectly determine the activation of ROCK-2. ROCK activity was determined by assaying the amount of phospho-Thr853 in the myosin phosphatase target subunit 1 (MYPT1) of myosin light chain (MLC) phosphatase. Western blot detection of p-MYPT1 and t-MYPT1(total MYPT1) were carried out to evaluate the activity of ROCK in the aortas. The membranes were then washed with PBS (pH 7.4, containing 0.1% Tween 20) and incubated with a horseradish peroxidase-conjugated goat anti-rabbit secondary antibody (1:5000, Amersham Biosciences, Piscataway, NJ, USA) for 2 h at room temperature. Finally, blots were visualized and quantified with an enhanced chemiluminescence (ECL) system (Fuji, Tokyo, Japan). All experiments were conducted at least three times. Relative light densities of the positive bands were calculated and expressed as a ratio to β-actin.

### Statistical analysis

Results are presented as the mean ± SE. For vasodilator responses studies, statistical analysis was carried out using two-way ANOVA tests followed by Bonferroni’s post hoc analysis. For other data, statistical analysis was carried out using a one-way ANOVA followed by Tukey's post hoc test. The significance level was set at 0.05. All analyses were carried out using SPSS 19.0 software.

## Results

### The fasudil has no effect on endothelial function

**Fig 1:**
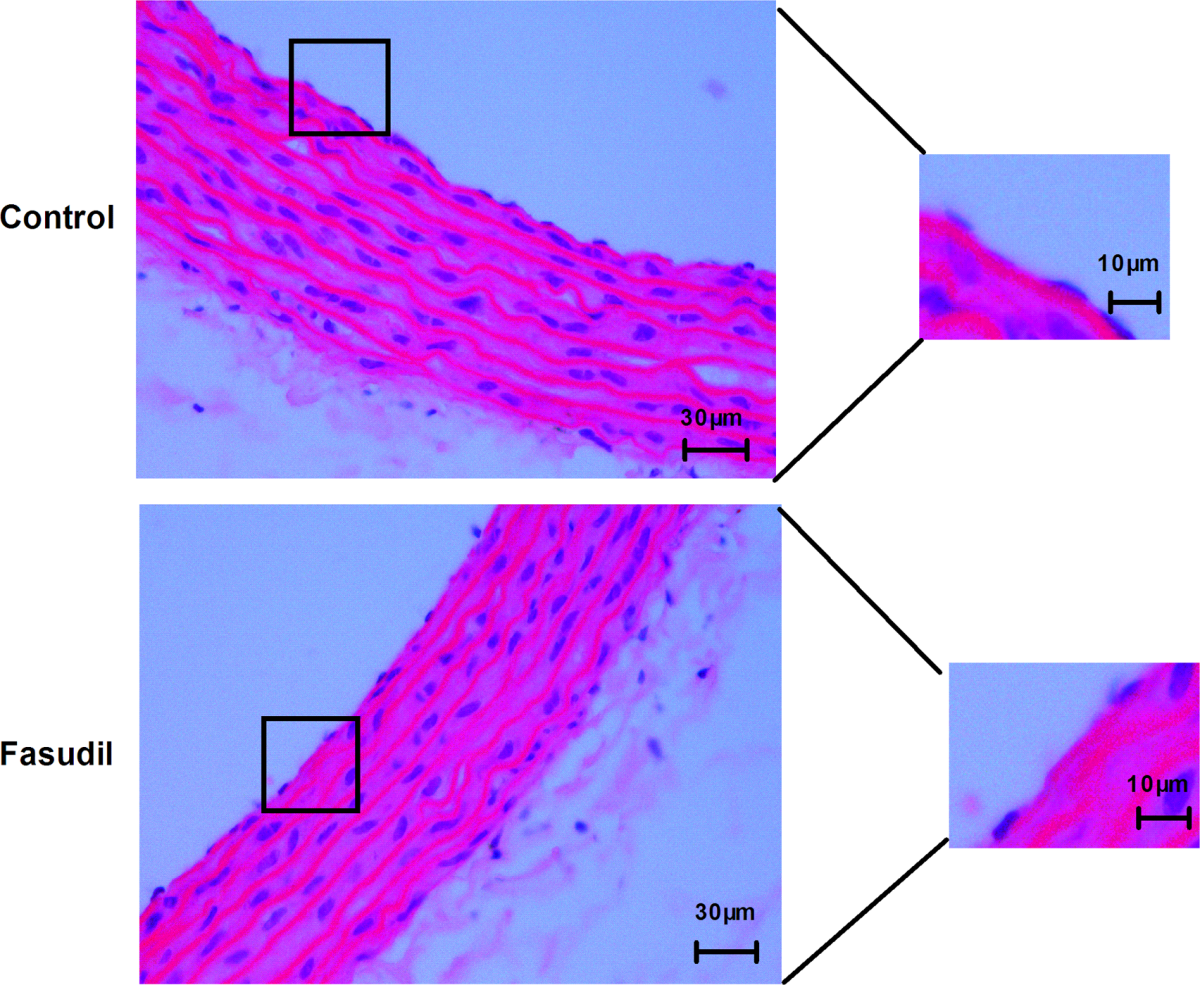
Fasudil has no effect on endothelial morphology of aorta. Representative microscopic photographs of aorta sections stained with H&E stain (scale bar = 30 μm; n = 6). The magnified image in the right.

Rats received fasudil (8 mg/kg, i.p.) did not show any effects on body weight, behaviour and mortality during the four-week observation period. There were no remarkable differences in related biochemical indicators, systolic blood pressures and heart rate (Table 1), and there were no obvious histological changes of the aorta in the fasudil group, compared to the control group (Fig 1).

**Table 1:** Effects of fasudil on blood biochemistry and body weight, systolic blood pressures, heart rate in normal rats.

Variables	Control group	Fasudil group
Initial BW (g)	200.81 ± 3.95	206.04 ± 4.67
Final BW (g)	237.67 ± 3.92	244.51 ± 4.81
Serum GLU (mmol/L)	6.46 ± 0.48	5.86 ± 0.35
Serum TC (mmol/L)	1.34 ± 0.05	1.37 ± 0.04
Serum TG (mmol/L)	0.55 ± 0.04	0.47 ± 0.03
Serum LDL (mmol/L)	1.09 ± 0.13	1.01 ± 0.11
Serum HDL (mmol/L)	0.62 ± 0.01	0.63 ± 0.02
Serum ALT (U/L)	37.03 ± 2.74	37.71 ± 2.25
Serum AST (U/L)	94.67 ± 3.88	93.98 ± 4.23
Serum CREA (μmol/L)	42.67 ± 3.22	38.68 ± 2.21
SBP (mmHg)	124.33 ± 4.94	123.33 ± 4.82
HR (bpm)	356.33 ± 19.37	356.17 ± 21.51

Control group: rats received normal saline solution; Fasudil group: rats received fasudil (8 mg/kg/day). BW: body weight. TC: total cholesterol. TG: triglyceride. LDL: low-density lipoprotein. HDL: high-density lipoprotein. ALT: alanine transferase. AST: aspartate transferase. CREA: creatinine. SBP: systolic blood pressures. HR: heart rate. Data were expressed as the mean ± SE (n = 6 in each group).

### Fasudil improved histopathological change of vascular endothelium in aortas

**Fig 2:**
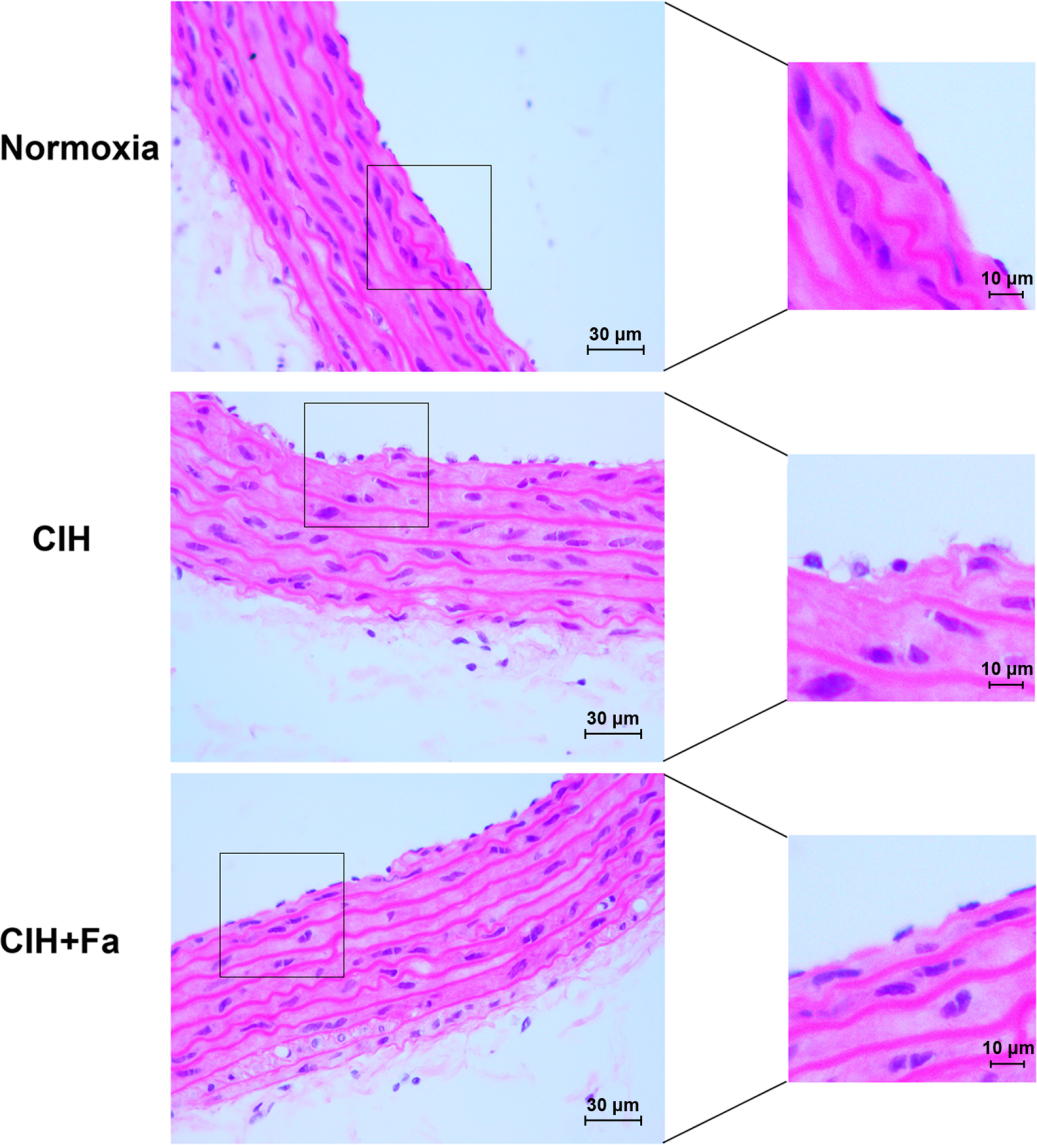
Histopathological changes in aortas when subjected to CIH. The aorta samples from different groups were analysed histochemically. Representative aorta histology in the Normoxia, CIH, and CIH + Fa groups were shown (magnification, 400 ×).

H&E stain presented the integrity of the vascular endothelium, consisting of an unbroken endothelial monolayer with regularly shaped and arranged endothelial cells in the Normoxia group (Fig 2). However, the endothelial layer exhibited remarkable histopathological changes in the CIH group, showing cellular oedema and partial exfoliation of endothelial cells (Fig 2). The histopathological change of vascular endothelium was improved in the CIH + Fa group compared with the CIH group (Fig 2).

### Fasudil improved vasodilator responses dysfunction in rats exposed to CIH

**Fig 3:**
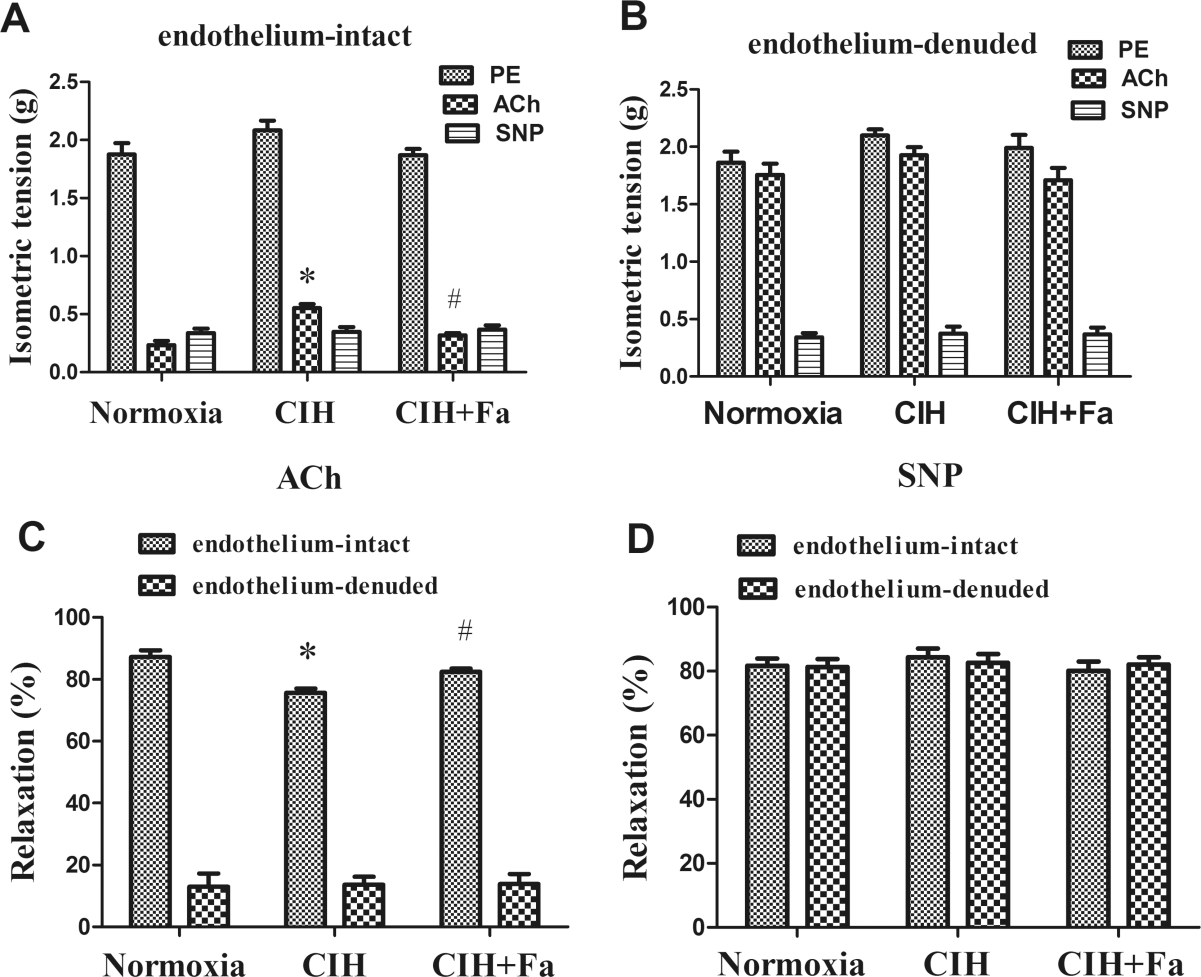
Vasodilator responses in endothelium-intact and endothelium-denuded aortas. (A) Results were expressed as an isometric tension in endothelium-intact rat aortas. (B) Results were expressed as an isometric tension endothelium-denuded in rat aortas. (C) Relaxation responses to ACh expressed as a percentage of PE-induced pre-contraction. (D) Relaxation responses to SNP expressed as a percentage of PE-induced pre-contraction. Values were the mean ± SE. * p < 0.05, CIH group vs Normoxia group; ^#^ p < 0.05, CIH + Fa group vs CIH group (n = 6 for each group).

To determine whether CIH affected endothelium-dependent or endothelium- independent vasodilation in rat aorta, we examined relaxation responses to ACh and SNP in endothelium-intact and endothelium-denuded aortas from rats in Normoxia, CIH and CIH + Fa group. The results showed that relaxation responses induced by ACh in the endothelium-intact CIH group decreased significantly compared with the Normoxia group and, fasudil significantly inhibited the decreased ACh-induced relaxation responses of the endothelium-intact CIH group (P < 0.05) (Fig 3A and 3C). However, ACh-induced relaxation responses showed no significant differences in all endothelium-denuded groups (Fig 3B and 3C). ACh-induced relaxation responses of all endothelium-denuded groups were significantly lower than that of all endothelium-intact groups (Fig 3C). The relaxation responses induced by SNP was not significantly different in all groups (Fig 3A and 3B and 3D).

### Fasudil increased NO in serum and aortic tissue in rats exposed to CIH

**Fig 4:**
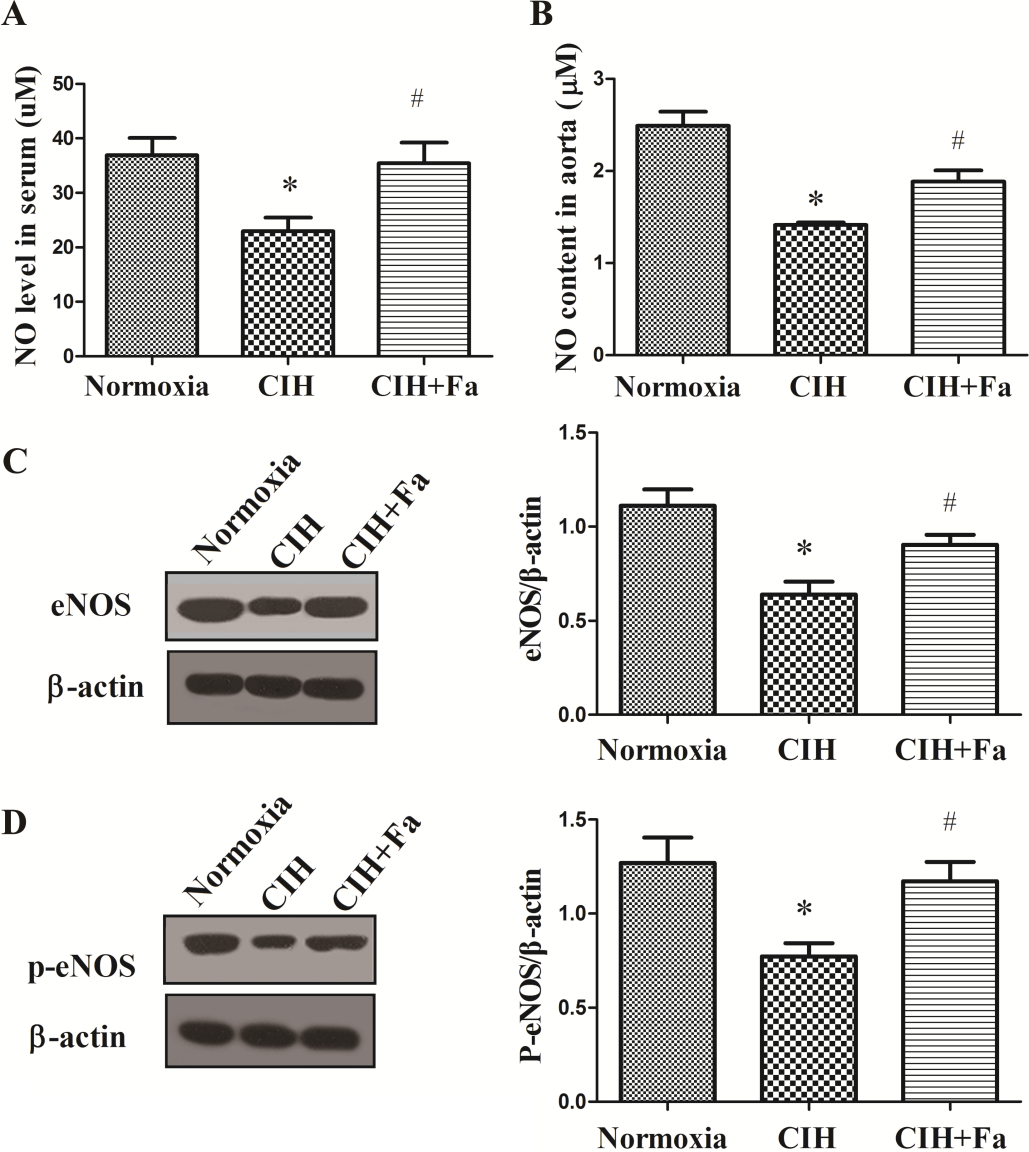
Levels NO in the serum and aorta. (A) NO content was measured in serum from the Normoxia, CIH and CIH + Fa groups by the Griess assay. (B) NO production was measured in aortas isolated from the Normoxia, CIH and CIH + Fa groups with a Griess assay. (C) eNOS protein was measured in aortas isolated from the Normoxia, CIH and CIH + Fa groups by Western blotting. (D) p-eNOS (Ser1177) protein was measured in aortas isolated from the Normoxia, CIH and CIH + Fa groups by Western blotting. The results were expressed as the mean ± SE. * p < 0.05, CIH group vs Normoxia group; ^#^ p < 0.05, CIH + Fa group vs CIH group (n = 6 for each group).

NO generated by endothelial cell played an important role in maintaining vascular microenvironment [22]. The total NO levels in serum and the aorta were dramatically reduced in CIH rats compared with the Normoxia group (*P* < 0.05), whereas the level of NO significantly increased in the CIH + Fa group compared with the CIH group (*P* < 0.05) (Fig 4A and 4B).

We also studied the effects of CIH on endothelial cell generating NO, and measured the marker of eNOS activity, eNOS (Ser1177) phosphorylation. The phosphorylation of eNOS (Ser1177) could accelerate NO production and dephosphorylation would decrease NO production [23]. As shown in Fig 4C and 4D, the levels of eNOS and p-eNOS (Ser1177) significantly decreased in CIH aortas compared with the Normoxia group, however, the levels of eNOS and p-eNOS were both increased with fasudil treatment (*P* < 0.05), comparing to CIH group.

### Fasudil decreased ET-1 in serum and aortic tissue in rats exposed to CIH

**Fig 5:**
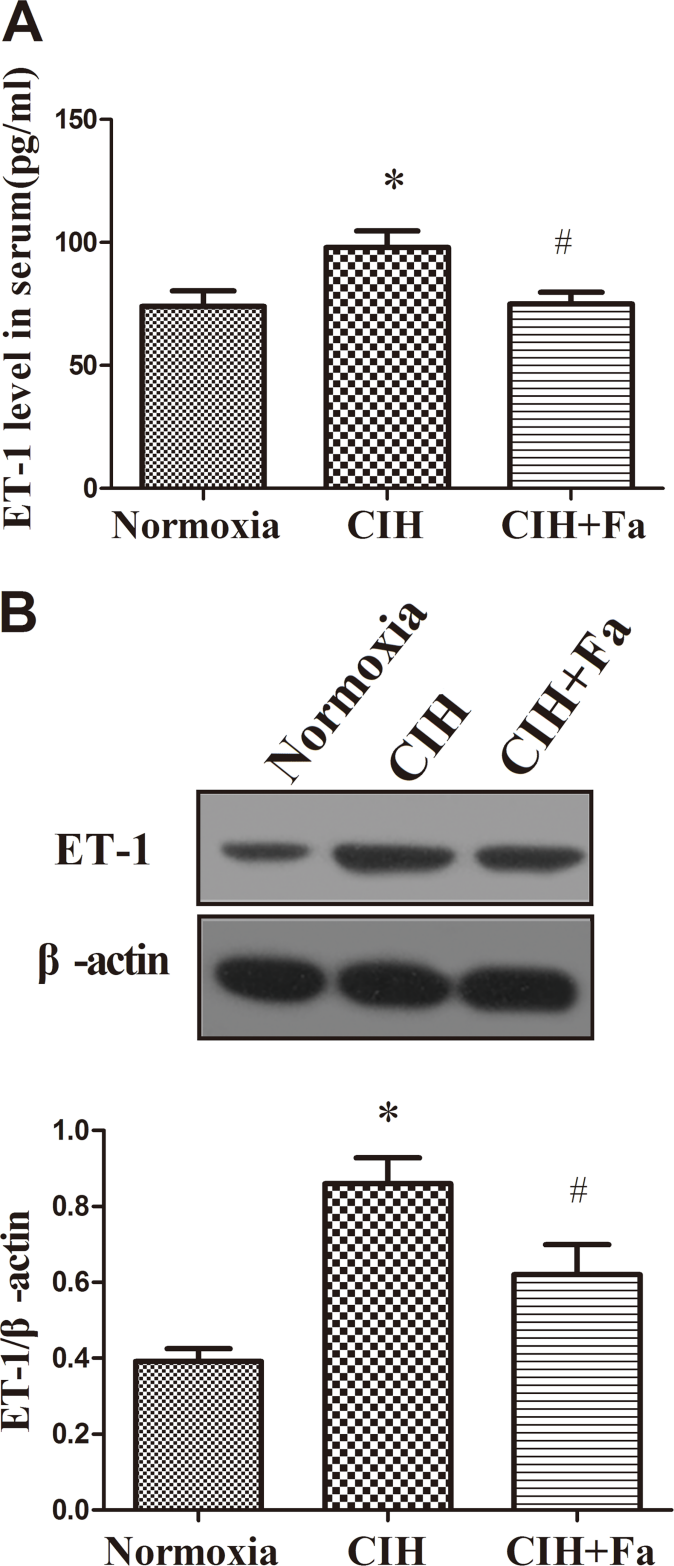
Levels ET-1 in the serum and aorta when subjected to CIH. (A) ET-1 content was measured in serum from the Normoxia, CIH and CIH+Fa groups by radioimmunoassay. (B) ET-1 protein levels were measured in aortas isolated from the Normoxia, CIH and CIH + Fa groups by western blot. The results were expressed as the mean ± SE. *p < 0.05, CIH group vs Normoxia group; ^#^ p < 0.05, CIH + Fa group vs CIH group (n = 6 for each group).

The level of ET-1 in serum showed a marked increase in the CIH group compared with the Normoxia group (Fig 5A), and western blot results revealed the ET-1protein level in aorta tissue also raised (Fig 5B). While, treatment with fasudil significantly prevented ET-1 increases in the serum and aorta tissue induced by CIH (P < 0.05) (Fig 5A and 5B).

### RhoA/ROCK/NFATc3 pathway mediated improving effect of fasudil on CIH

**Fig 6:**
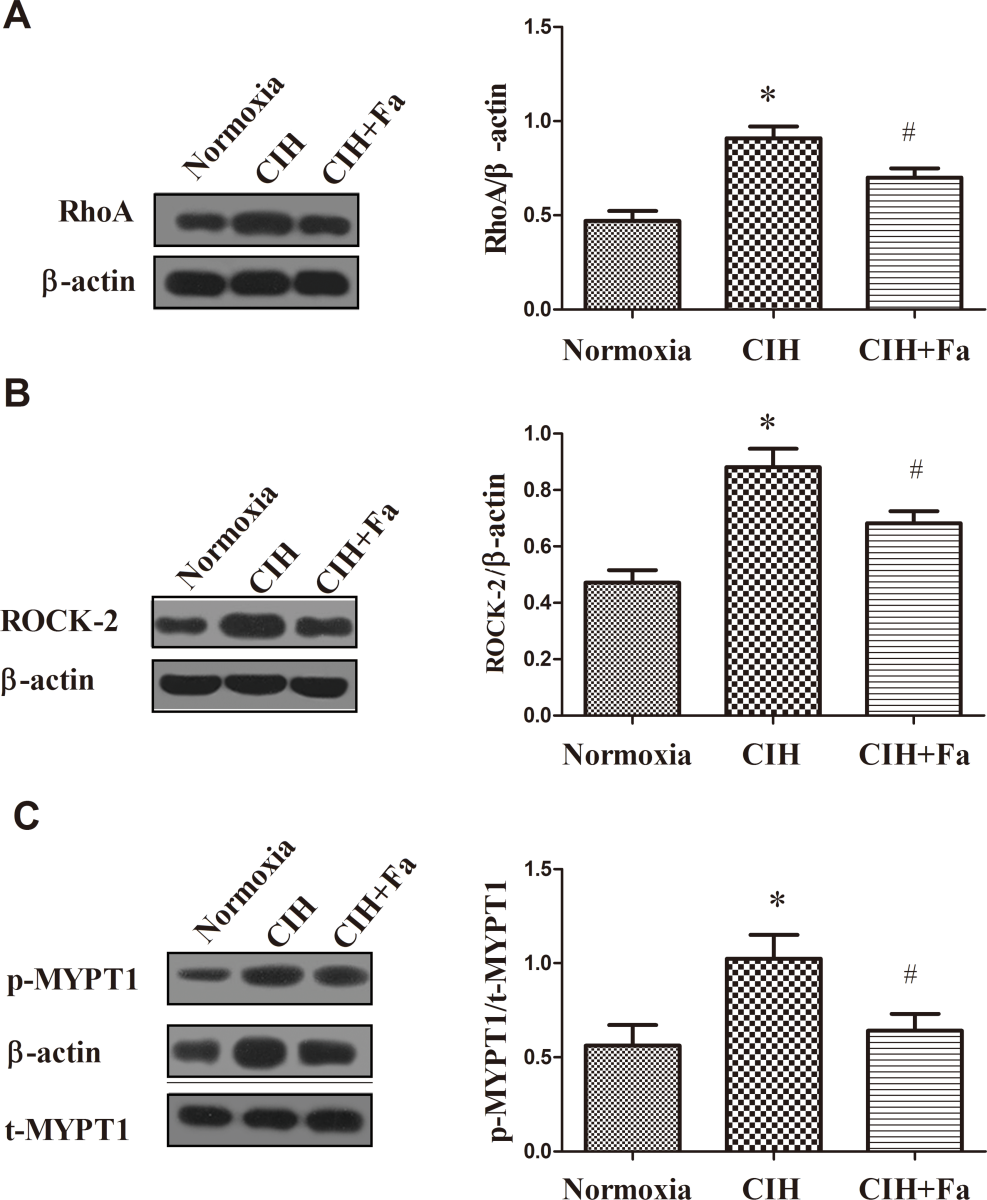
Expression of RhoA, ROCK-2, p-MYPT1, and t-MYPT1 proteins in aortas when subjected to CIH. (A-B) RhoA and ROCK-2 protein levels were measured in aortas from Normoxia, CIH and CIH + Fa groups by Western blot. (C) p-MYPT1 (Thr853) and t-MYPT1 protein levels were measured in aortas isolated from the Normoxia, CIH and CIH + Fa groups by Western blotting. The results were expressed as the mean ± SE. * p < 0.05, CIH group vs Normoxia group; ^#^ p < 0.05, CIH + Fa group vs CIH group (n = 6 for each group).

However, which signal pathway did regulate the level of NO in the endothelium-intact aortas during CIH? We firstly detected the RhoA protein level in the aortas tissue. Fig 6A showed the level of RhoA was higher in CIH group (P < 0.05) than that in the Normoxia group. And the level of ROCK-2 was also elevated (*P* < 0.05) in the CIH group (Fig 6B). While, fasudil treatment could decrease the expression of RhoA and ROCK-2 protein, comparing to the CIH group (Fig 6A and 6B).

As shown in Fig 6C, p-MYPT1/t-MYPT1 was significantly elevated in the CIH group compared with the Normoxia group (*P* < 0.05). However, treatment with fasudil significantly decreased p-MYPT1/t-MYPT1 compared with the CIH group (Fig 6C).

**Fig 7:**
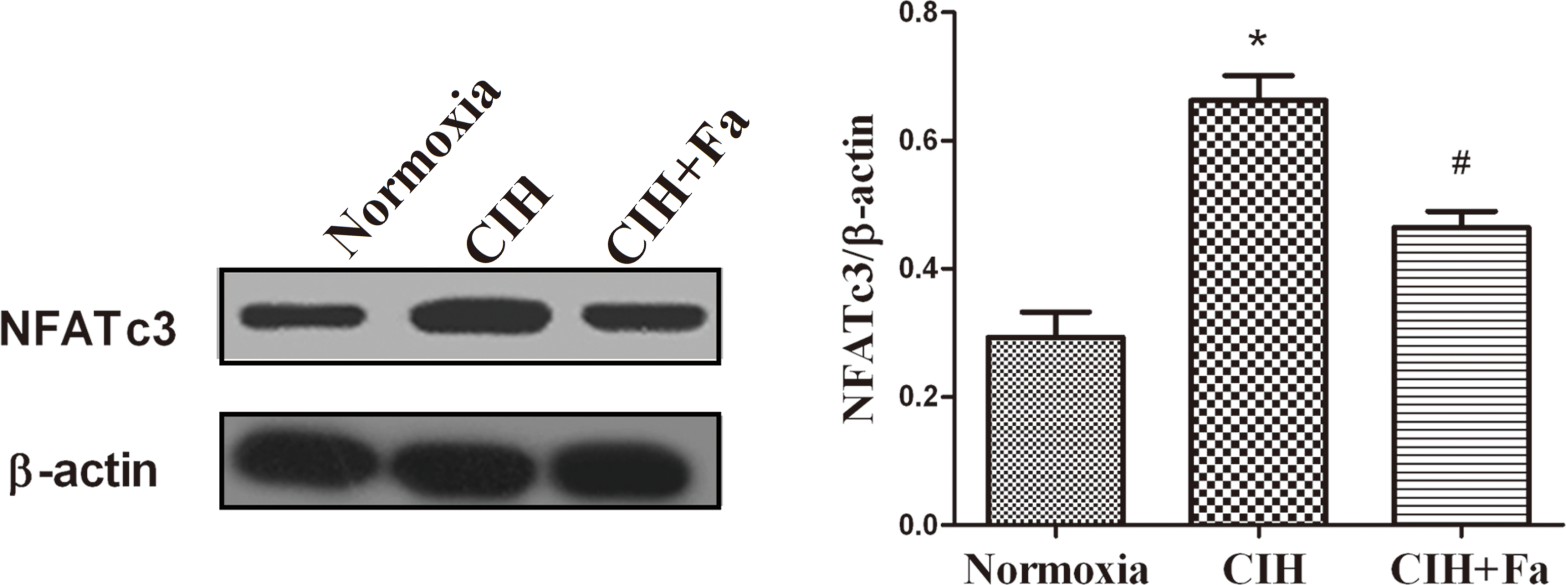
Expression of NFATc3 protein in aortas when subjected to CIH. NFATc3 protein levels were measured in aortas from Normoxia, CIH and CIH + Fa groups by Western blotting. The results were expressed as the mean ± SE. * p < 0.05, CIH group vs Normoxia group; ^#^ p < 0.05, CIH + Fa group vs CIH group (n = 6 for each group).

NFATc3 is considered a downstream substrate of ROCK and may be involved in CIH-induced endothelial dysfunction. Results showed that the expression of NFATc3 protein increased in the CIH group compared with the Normoxia group (P < 0.05). Treatment with fasudil inhibited the increases of expression of NFATc3 compared with the CIH group (Fig 7).

## Discussion

OSA, a worldwide sleep-breathing disease, is known as an independent dangerous factor for cardiovascular diseases [24]. OSA elicited CIH, which contributed to endothelial dysfunction and cardiovascular diseases [25]. In the present study, a CIH rat model was established by simulating the OSA state. The results of our study showed that CIH-induced endothelial dysfunction associated with increased ET-1 and decreased NO in rat aorta. Fasudil attenuated endothelial dysfunction induced by CIH through inhibiting ROCK activation. Thus, RhoA and ROCK activity played an important role in the pathogenesis of CIH by mediating a potent vasoconstrictor response. Furthermore, we demonstrated that increased RhoA/ROCK/NFATc3 pathway and ROCK activity were associated with a functional decrease in endothelium dependent vasodilation in aortas, which contributes to the pathogenesis of CIH-induced dysfunction of endothelium.

The vascular endothelium plays an important role in the regulation of various vascular functions and homeostasis [26]. The damage and/or malfunction of the artery endothelium might be associated with various cardiovascular diseases. Endothelial dysfunction is considered an early marker of vascular abnormalities before clinically obvious cardiovascular disease [27–29]. Damage to the artery endothelium might cause abnormal release of vasoactive factors, disrupting the balance of its own regulation system, such as up-regulating ET-1 levels and down-regulating NO production.

In the past few decades, a large body of evidence had indicated that the endothelial cell was capable of releasing vasoactive substances. The imbalance between ET-1 and NO levels of serum greatly contributed to the risk of cardiovascular diseases [30]. Previous studies have shown that vascular endothelial dysfunction occurred in the CIH model prior to the development of cardiovascular diseases, which suggested that systematic endothelial dysfunction was the starting phase of cardiovascular diseases by CIH [31]. Our study provided direct evidence of vascular endothelial dysfunction in CIH rats. NO content was significantly lower and ET-1 levels were significantly higher in CIH rats. These changes was improved by treatment with fasudil. The results suggested that vascular endothelial dysfunction was the earliest cardiovascular abnormality in OSA and contributed to the subsequent development or progression of OSA-related cardiovascular disease [32].

Both ACh and SNP are common vasodilators. It is well known that vasodilatation caused by ACh involves the release of endothelium-derived NO, and relaxation caused by SNP does not involve NO [21]. Previous studies have shown that the vascular reaction to ACh was mediated by NO released from the vascular endothelium of skeletal muscle and cerebrum of rats [32], and it was reported that the ACh-induced vasodilatation in two types of arteries was damaged in CIH [33]. Study showed that even mild OSA was accompanied by decreased endothelium-dependent vascular dilation [34]. NO, as a main vasodilator, was synthesized in the vascular endothelium and decreased in the plasma of patients with OSA [35, 36]. It has been shown that endothelial dysfunction induced by CIH is a systemic pathological condition of the vascular endothelium, not just the peripheral vascular but also the aorta. Aortic endothelial dysfunction could promote the occurrence of cardiovascular events in OSA patients [37, 38]. To evaluate whether CIH affected endothelium-dependent vasodilation function, vasodilator responses to SNP and ACh were examined in the aorta. Our results showed that CIH exposure impaired endothelium-intact relaxation responses to ACh, and fasudil significantly alleviated the impaired ACh-induced relaxation responses. However, CIH exposure did not affect endothelium-intact aortic vasodilator responses to SNP. These data implied that endothelium-derived vasodilation was impaired by CIH. In addition, these results further supported that ROCK involved with the CIH-induced endothelial dysfunction.

As an important biomarker of endothelial function, NO was synthesized from its precursor L-Arginine by a family of NOS. eNOS might mediate endothelial NO generation and release [39]. Production of NO in endothelial cells by eNOS was modulated by phosphorylation of eNOS, and eNOS ser1177 phosphorylation leads to NO production increases. Down-regulation of vascular eNOS and reduced activation of eNOS were characteristic of vascular endothelial dysfunction [22]. NFAT could play a potential role in endothelial dysfunction and inhibition eNOS [40]. A previous study showed that NFATc3 might contribute to arterial remodeling associated with hypoxia-intermittent hypoxia [41], and NFAT was a novel mechanism causing endothelial dysfunction under hyperglycaemia [42]. In the present study, exposure to CIH reduced the generation of NO, inhibited eNOS protein expression, reduced eNOS activation and increased NFATc3 protein expression in rat aortas, which were improved by fasudil treatment. These data suggested that increased NFATc3 expression played a role in CIH-induced endothelial dysfunction. Therefore, it is possible that ROCK is associated with NFATc3/eNOS pathway in the CIH condition.

**Fig 8:**
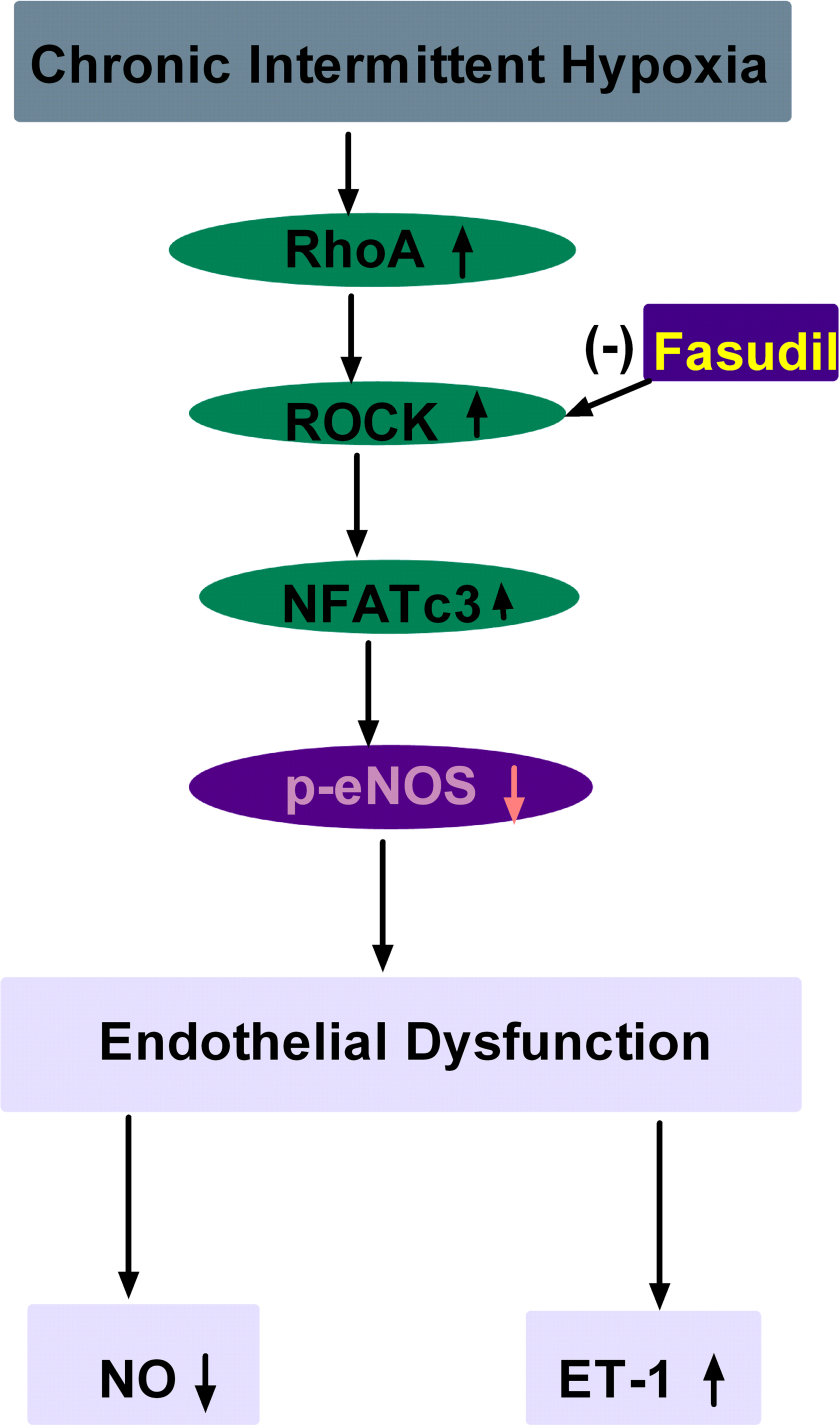
Schematic illustration of RhoA/ROCK/NFATc3 pathway contributing to endothelial dysfunction induced by CIH. CIH increased RhoA, ROCK and NFATc3 protein expression in aortas, and decreased p-eNOS protein expression, which lead to endothelial dysfunction. Fasudil could improve these changes.

Studies demonstrated that RhoA/ROCK activation played an important role in various cardiovascular diseases [43], and acted as a convergent node in the pathogenesis of vascular diseases [44]. Inhibition of this signalling pathway could reduce the risk of adverse cardiovascular events and provided pharmacological tools for vascular studies [45–49]. More evidence showed that eNOS expression and activity were regulated by RhoA/ROCK [50], and RhoA/ROCK negatively regulated eNOS (Thr495) and eNOS (Ser1177) and decreased vasodilation [26]. RhoA/ROCK decreased eNOS expression through down-regulation of eNOS mRNA stability, and decreased eNOS activity through inhibition of eNOS phosphorylation at Ser1177 via the PI3-kinase/Akt pathway and acceleration of eNOS phosphorylation at Thr495 [51]. In hypertensive profilin1 transgenic mice, activation of the RhoA/ROCK pathway significantly inhibited eNOS expression and phosphorylation (Ser1177) in the mesenteric arteries [52]. ROCK blockers, which block ROCK activity, can prolong the eNOS mRNA biological half-life and increase eNOS expression in vascular disease. MYPT1 is a major downstream target of ROCK. In recent studies, measurement of p- MYPT/t- MYPT was used as an indirect method for assessing ROCK activity [53]. Thus, MYPT and p-MYPT proteins were measured to indirectly determine the activation of ROCK-2 in our study. Our results showed that CIH significantly elevated p-MYPT1/t-MYPT1 and increased RhoA and ROCK protein expression. Previous study has showed that ROCK inhibition prevents intermittent hypoxia-induced NFATc3 activation in mouse mesenteric arteries both in vivo and ex vivo [41]. Our results demonstrated that CIH up-regulated NFATc3 expression in rat aorta arteries, which was dependent on RhoA/ROCK pathway. Acting as the downstream target of RhoA/ROCK, whether NFATc3 was involved in the regulation of eNOS expression via the RhoA/ROCK pathway was not clear. Our study showed that CIH increased RhoA/ROCK-2/NFATc3 protein expression and ROCK-2 activation, and inhibited eNOS expression and phosphorylation (Ser1177), and reduced NO production. The results suggested that the pathway of RhoA/ROCK/NFATc3 contributed to endothelial dysfunction by CIH. In the present study, inhibition of the RhoA/ROCK/NFATc3 pathway by fasudil in CIH rats increased eNOS and NO levels, and decreased ET-1 levels and maintained the balance of ET-1 and NO. These data further suggested that the RhoA/ROCK/NFATc3 pathway could mediate endothelial dysfunction by CIH in aortas (Fig 8).

## Conclusions

In conclusion, this study demonstrates that CIH-induced endothelial dysfunction in OSA is mediated by eNOS/NO reduction through RhoA/ROCK/NFATc3 pathway activation. ROCK inhibited by fasudil significantly improves CIH induced endothelial dysfunction in rats. Thus, fasudil might be a feasible therapeutic option to the progression to cardiovascular diseases in OSA.
